# Signet Ring Cell Colorectal and Appendiceal Cancer: A Small Signet Ring Cell Component Is Also Associated with Poor Outcome

**DOI:** 10.3390/cancers15092497

**Published:** 2023-04-26

**Authors:** Malin Enblad, Péter Pál Egerszegi, Helgi Birgisson, Tobias Sjöblom, Bengt Glimelius, Joakim Folkesson

**Affiliations:** 1 Department of Surgical Sciences, Colorectal Surgery, Uppsala University, 751 85 Uppsala, Sweden; 2 Department of Immunology, Genetics and Pathology, Clinical Pathology, Uppsala University, 751 08 Uppsala, Sweden; 3 Department of Immunology, Genetics and Pathology, Experimental and Clinical Oncology, Uppsala University, 751 85 Uppsala, Sweden

**Keywords:** signet ring cells, colorectal cancer, appendiceal cancer, peritoneal metastases

## Abstract

**Simple Summary:**

Signet ring cell (SRC) carcinoma of colorectal and appendiceal cancer is rare but is recognized as the histopathological subtype with the poorest prognosis. However, the prognostic relevance of a SRC component <50% is unclear. The aim of this study was to provide a clinicopathological characterization of all SRC-containing colorectal and appendiceal cancers, including those with <50% SRCs. The results showed that SRCs, both ≥50% and <50%, were associated with aggressive histopathological features, advanced stages, and, particularly, peritoneal metastases. Information about the presence of SRCs in tumour tissue, not only in the case of ≥50% SRCs, should be routinely registered in pathology reports and clinical registers to enable larger studies that can aid our understanding of SRCs in colorectal and appendiceal cancers.

**Abstract:**

Background: Colorectal signet ring cell (SRC) carcinoma with ≥50% SRCs (SRC ≥ 50) has a poor prognosis, but the prognostic role of SRCs < 50% (SRC < 50) is unclear. The aim of this study was to provide a clinicopathological characterization of SRC colorectal and appendiceal tumours and analyse the importance of the SRC component size. Methods: All patients in the Swedish Colorectal Cancer Registry diagnosed with colorectal or appendiceal cancer in 2009–2020 at Uppsala University Hospital, Sweden, were included. The SRCs were verified, and the components estimated by a gastrointestinal pathologist. Results: Of the 2229 colorectal cancers, 51 (2.3%) had SRCs, with a median component size of 30% (interquartile range of 12.5–40) and 10 (0.45%) had SRC ≥ 50. The SRC tumours were primarily localized in the right colon (59%) and appendix (16%). No patients with SRCs had stage I disease, and 26 (51%) had stage IV, of whom, 18 (69%) had peritoneal metastases. The SRC tumours were often high grade with perineural and vascular invasion. The 5-year overall survival (OS) rate for patients with SRC ≥ 50 were 20% (95% confidence interval (CI) 6–70), for SRC < 50, 39% (95% CI 24–61); and for non-SRCs, 55% (95% CI 55–60). Among the patients with SRC < 50 and <50% extracellular mucin, the 5-year OS was 34% (95% CI 19–61), while those with ≥50% extracellular mucin had an OS of 50% (95% CI 25–99). The 5-year recurrence-free survival rates were 51% (95% CI 13–83) for patients with SRC tumours, as compared to 83% (95% CI 77–89) and 81% (95% CI 79–84) for mucinous and non-mucinous adenocarcinoma, respectively. Conclusions: The presence of SRCs was strongly associated with aggressive clinicopathological features, peritoneal metastases, and poor prognosis, also when they make up <50% of a tumour.

## 1. Introduction

Signet ring cell (SRC) carcinoma is a distinct clinical and histopathological subtype of colorectal cancer, with aggressive behaviour. It was first described as the primary linitis plastica type of colorectal cancer [1], currently referred to as poorly cohesive gastric cancer [2]. In colorectal cancer, SRCs have been associated with right-sided colon tumours as well as large, advanced tumours and frequent peritoneal metastasis [3,4,5,6,7,8]. SRC carcinoma is rare, with a reported prevalence of 0.6–1.1% [3,4,6,8,9,10,11,12], but its clinical relevance is recognized, as it is the histopathological subtype with the poorest prognosis [3]. In addition, it is more prevalent in early onset colorectal cancer (often defined as <50 years) [3,4,6,7,12,13,14], which is a group with rising incidence [15,16].

SRCs have intracellular mucin pools dislodging the nucleus to the cell periphery, giving it the typical appearance of a signet ring. By definition, the tumour mass of a proper SRC carcinoma has ≥50% SRCs (SRC ≥ 50) [2]. The poor prognosis associated with SRCs has been attributed to the histopathological high-grade features, advanced tumour stage, and frequent lymph node and distant metastasis, especially peritoneal metastasis, but SRC ≥ 50 has also been demonstrated as an independent, poor prognostic factor [3,4,7,8]. A few studies have analysed the prognostic relevance of an SRC component of <50% (SRC < 50), and the results have indicated that these patients have as poor a prognosis as those with SRC ≥ 50, or at least, worse than those with ordinary adenocarcinoma (AC) [7,17,18,19,20,21].

Although clinicians have recognized the presence of SRCs as an adverse prognostic factor and studies have indicated the unique biological behaviours of these tumours [22,23], the treatment and follow-up strategies for SRC carcinoma do not differ from those for AC or mucinous adenocarcinoma (MAC). Therefore, the aim of this study was to provide a clinicopathological characterization of SRC colorectal tumours and analyse the prognostic role of SRC < 50. In addition, this study also included adenocarcinomas of the appendix, where SRC tumours seem to be more common than in the colon or rectum, though this has yet to be confirmed.

## 2. Materials and Methods

### 2.1. Study Population

All patients diagnosed with a colorectal or appendiceal cancer (only adenocarcinoma), between 2009 and 2020 at Uppsala University Hospital in Sweden, were included, and the data were retrieved from the Swedish Colorectal Cancer Registry (SCRCR) [24], a national quality registry for colorectal cancer. In cases of synchronous or metachronous colorectal cancer, the most advanced tumour was included in the analyses. The SCRCR contained no information about SRCs, and using the hospital’s data system, patients with SRCs were identified by a free-text search for ‘signet’ in the histopathology reports. The identified reports were then reviewed for relevance, and re-examinations of the histopathological sections were performed to confirm the presence of SRCs. The SRCs were identified in the pre-operative biopsy specimen, the surgical resection specimen, and/or the metastatis specimen. Patients with SRC tumours were then identified in the SCRCR using their personal identification numbers [25].

### 2.2. Management of Colorectal Cancer

All patients were managed in accordance with national guidelines, which included preoperative staging with computed tomography of the abdomen and thorax, high-resolution magnetic resonance imaging of the rectum in rectal cancer, and discussion at a multidisciplinary team conference. Staging; surgical treatment, including cytoreductive surgery and hyperthermic intraperitoneal chemotherapy, resection of liver and lung metastases; radiotherapy; chemotherapy; and radio-chemotherapy were all performed at Uppsala University Hospital. Routine follow-up comprised abdomino-thoracic computed tomography and carcinoembryonic antigen testing after one and three years. A colonoscopy was scheduled five years after surgery. Stage IV colorectal cancer patients treated with curative intent and radical surgery were followed up every sixth month for two years and after three years. Medical treatment of metastatic disease largely followed the European Society for Medical Oncology guidelines [26]. Patients with SRC ≥ 50 or SRC < 50 were not treated differently from those with ordinary colorectal cancer, and according to the national Swedish guidelines and the National Comprehensive Cancer Network [27], the presence of SRCs was not considered a risk factor when deciding whether adjuvant treatment should be recommended or not.

### 2.3. Histopathology

Preoperative biopsy, primary tumour resection, and metastasis resection specimens were routinely fixed in 4% buffered formaldehyde and embedded in paraffin (FFPE). The 4 µm sections were stained with haematoxylin and eosin and re-examined by an experienced gastrointestinal pathologist (P.P.E). The SRCs had a typical appearance, resembling a signet ring, with intracellular mucin pools dislodging the nucleus to the cell periphery. Tumours with ≥50% neoplastic cells with SRC morphology were classified as proper SRC carcinomas, SRC ≥ 50, and tumours with <50% SRCs as SRC < 50, in accordance with the World Health Organization (WHO) classification [2]. In this study, the proportions of SRCs in the tumour tissue were examined, and all SRC-containing tumours were included. SRC-containing colorectal tumours sometimes have extracellular mucin content; which was also re-examined. In addition, neuroendocrine features (immunohistochemistry of chromogranin and synaptophysin) were noted. For subgroup analyses of clinical characteristics and survival, the patients were grouped into 5 groups based on histopathological characteristics: AC, MAC, Group 1 Signet (SRC ≥ 50, irrespective of extracellular mucin component); Group 2 Signet (SRC < 50 and extracellular mucin component <50%); and Group 3 Signet (SRC < 50, and extracellular mucin component ≥50%).

### 2.4. Clinicopathological Characteristics

Differences in baseline characteristics between SRC-containing and non-SRC-containing tumours were analysed using SCRCR data. The SCRCR contained information about baseline characteristics, preoperative staging, surgical treatment, histopathological classification, oncological treatments, and follow-up. The tumour node metastatis (TNM) stage was reported in accordance with the 8th edition of the International Union Against Cancer’s TNM Classification on Malignant Tumours [28]. In the present study, staging was reported using the pathological TNM stage when available. In patients with inoperable/palliative disease or undergoing preoperative oncological treatment, the clinical TNM stage was used. The pattern of metastasis was described using the results of preoperative staging and complemented in cases of preoperatively discovered metastases. Histopathological information was reported to the registry in accordance with the WHO classification of tumours of the digestive system, and tumours were considered mucinous in cases of ≥50% extracellular mucin [2]. Follow-up information was registered at three and five years, or at the time of recurrence or death. More detailed descriptions of the patients with SRC tumours were obtained from medical reports.

### 2.5. Statistical Analyses

Continuous data were presented as medians with interquartile ranges (IQRs). The Chi-squared test and Fisher’s exact test were used for comparisons of the categorical data and the Mann–Whitney U test was used to compare continuous data. Overall survival (OS) and recurrence-free survival (RFS) rates were calculated from the date of diagnosis to death or recurrence, respectively, using Kaplan–Meier curves. The cumulative proportions of the 5-year OS and RFS were presented with 95% confidence intervals (95% CIs). Differences in survival rates were analysed using the log-rank test. Risk factors for recurrence were analysed for stage I–III patients undergoing resection surgery, using univariable and multivariable Cox proportional hazard regression, and were presented as hazard ratios (HRs) with 95% CIs. Risk factors were analysed only for colon and appendiceal cancer, as there was only one case of SRC rectal cancer undergoing resection. Significant variables in the univariable analyses were included in the multivariable analysis. A *p*-value lower than 0.05 was considered statistically significant. R version 4.2.2 (R foundation for Statistical Computing, Vienna, Austria) was used for data management and statistical analyses.

## 3. Results

### 3.1. Study Population

Between 2009 and 2020, 2229 patients were diagnosed with colorectal cancer (including appendiceal cancer) in Uppsala, Sweden, according to the SCRCR. SRCs were present in the tumour tissue in 51 (2.3%) patients, according to the histopathology reports, and confirmed after re-examination of the histopathological sections. Of these, 10 (0.45% of all) had a SRC proportion of ≥50% in any of the examined sections (primary tumour or metastasis).

### 3.2. Histopathological Review

**Figure 1:**
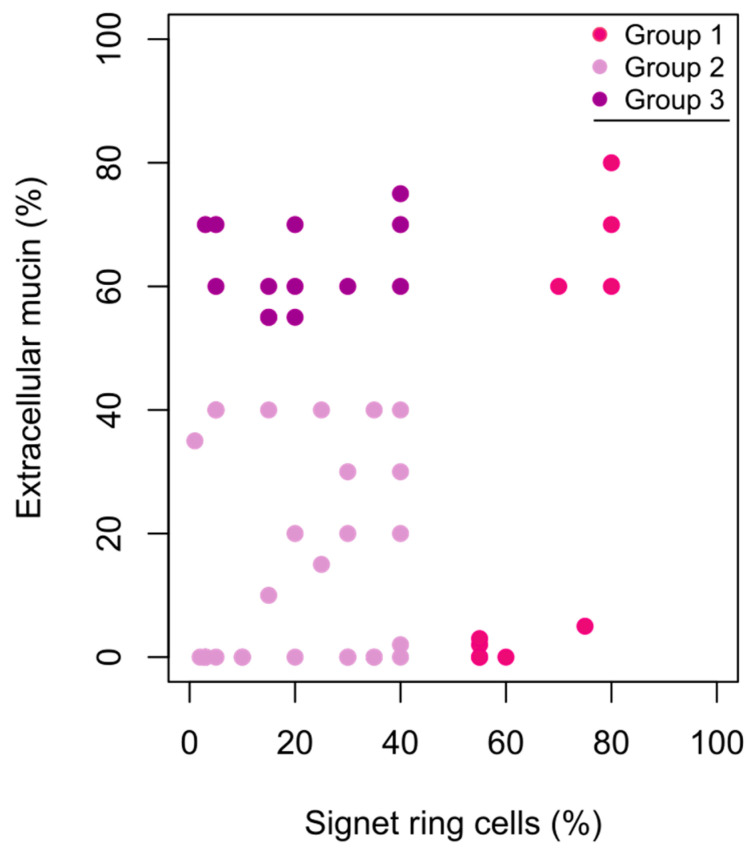
Scatterplot of signet ring cell component (%) and extracellular mucin component (%) in colorectal and appendiceal cancer (*n* = 51). Group 1 ≥ 50% signet ring cells, any extracellular mucin component; Group 2 ≤ 50% signet ring cells, <50% extracellular mucin; Group 3 ≤ 50% signet ring cells, ≥50% extracellular mucin.

The histopathological review of the specimens confirmed the presence of SRCs. The proportions of SRCs in the tumours were 1–80%, with a median of 30% (IQR 12.5–40). The majority of the tumours had an extracellular mucin component. Based on the histopathological review, the patients were divided into three groups depending on the SRC and extracellular mucin components (Figure 1). The SRC and extracellular mucin components were generally based on the primary tumour’s FFPE tissue (*n* = 39, 76%). Metastatis FFPE tissue (*n* = 7, 14%) and primary tumour biopsy FFPE tissue (*n* = 5, 10%) were used for patients who never underwent resection of the primary tumour. SRC ≥ 50 were represented in all three types of FFPE tissue.

One tumour originating in the transverse colon was classified as a mixed adenoneuroendocrine carcinoma and had a 15% SRC component. A total of 5 tumours originating in the appendix or the caecum had minor neuroendocrine features and SRC components of 5–55%.

### 3.3. Clinicopathological Characteristics

**Figure 2:**
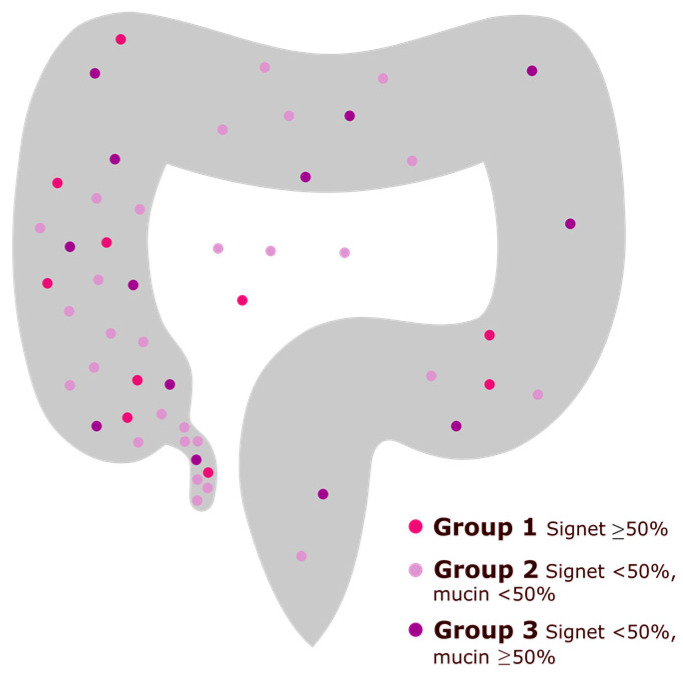
The primary tumour localization of signet ring cell colorectal and appendiceal cancer. Colours represent histopathological characteristics of the primary tumour (signet ring cell component or extracellular mucin component). In four patients, the exact localization of the primary tumour was unknown.

Based on the data from the SCRCR, the clinical characteristics of patients with and without SRCs in the tumours were compared (Table 1). There were no differences in median age or sex distribution. The SRC tumours primarily originated from the right colon and were uncommon in the rectum. The appendix was the primary tumour localization in 16% of the SRC tumours and 0.5% of the non-SRC tumours. A total of 70% of the SRC ≥ 50 tumours were localized in the right colon (not including the appendix, see Figure 2). At the time of diagnosis, 51% of patients with SRCs had stage IV disease, and 0% had stage I disease. Emergency surgery (resection or diversion) was performed in 22% of patients with SRC tumours and 11% of patients with non-SRC tumours (Table 1). The clinical characteristics for the different SRC groups are shown in File S1 Table S1a. The median age was lower among patients with SRC ≥ 50, 66 years (IQR 63–70.0), and 60% were male. A total of 60% of patients with SRC ≥ 50 had stage IV disease while the rest had stage III disease.

Based on the additional information retrieved from the medical records of the patients with SRC tumours, one had inflammatory bowel disease, and two had confirmed hereditary colorectal cancer. A total of 10 (20%) patients had a history of previous other malignancy (uterine *n* = 3, breast *n* = 3, prostate *n* = 1, myeloma *n* = 1, lung *n* = 1, testicular *n* = 1). Primary tumour resection had been performed in all stage I–III patients and in 13 (50%) of the stage IV patients. Of the stage IV patients undergoing resection, 7 underwent metastasis surgery with curative intent (cytoreductive surgery and hyperthermic intraperitoneal chemotherapy = 6, liver surgery = 1), and 1 cytoreductive surgery patient was alive and recurrence-free after 2.8 years.

The differences in the histopathological characteristics between SRC and non-SRC tumours are shown in Table 2. Rectal cancer was not analysed since there were only two SRC rectal tumours, of which only one had been resected. No SRC tumours were pT1/pT2, and most were pN2 nodal stage. Vascular and perineural invasion, high-grade differentiation, and extracellular mucin were all more common in SRC tumours (*p* < 0.001). The histopathological characteristics were generally similar for the different SRC groups, except that no patient with SRC ≥ 50 had pN0 stage (File S1 Table S1b).

### 3.4. Pattern of Metastasis

**Figure 3:**
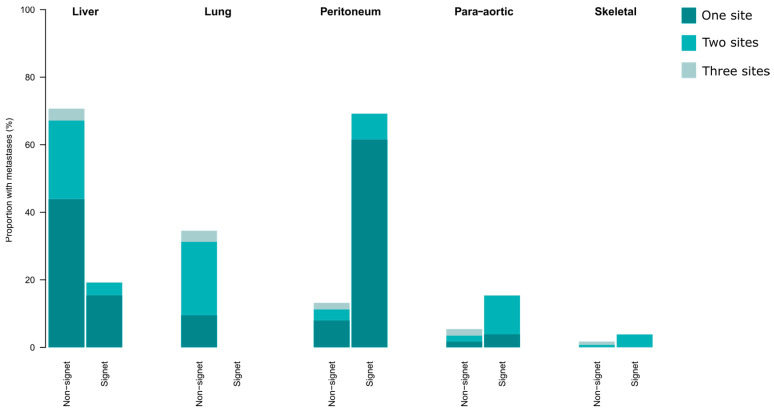
The sites of synchronous metastases in patients with stage IV colorectal and appendiceal cancer, separated into signet ring cell tumours (*n* = 26) and non-signet ring cell tumours (*n* = 515). The colour grading indicates if patients had one or multiple sites of metastases. In cases of multiple sites, the patient was included in two or more sites.

Synchronous metastases were more common in patients with SRC tumours (*n* = 26, 51%) than in patients with non-SRC tumours (*n* = 515, 24%). The 5 most common sites of metastases in patients with SRC and non-SRC tumours, respectively, are shown in Figure 3. Of all the patients with SRC tumours, 35% had peritoneal metastases at time of diagnosis. Among the patients with SRC tumours and stage IV disease, 69% had peritoneal metastases, and 62% had the peritoneum as the only site of metastasis. No patients with SRC tumours had lung metastases. Para-aortic lymph-node metastases were more common in the patients with SRC tumours.

### 3.5. Overall Survival

**Figure 4:**
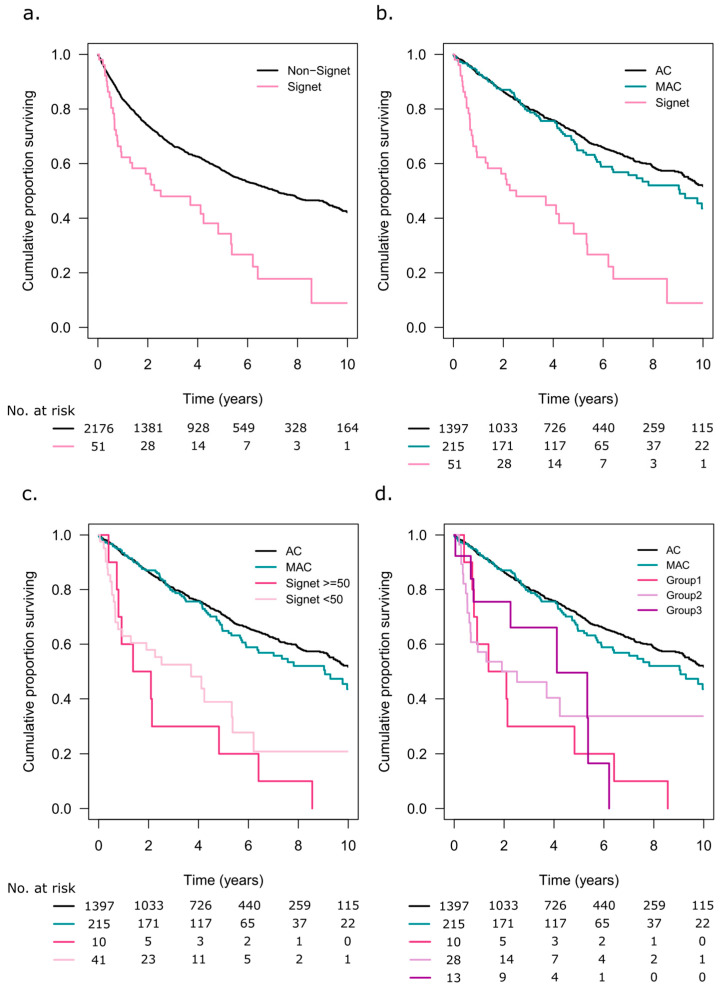
(**a**) Overall survival (OS) for patients with signet ring cell tumours and non-signet ring cell tumours. (**b**) OS in patients with adenocarcinoma (AC), mucinous adenocarcinoma (MAC), and signet ring cell tumours. Only patients with information on extracellular mucin were included. (**c**) OS in patients with adenocarcinoma (AC), mucinous adenocarcinoma (MAC), and signet ring cells ≥50% or <50% of the tumour tissue. (**d**) Signet ring cell tumour patients separated based on the extent of signet ring cells and extracellular mucin: Group 1 ≥ 50% signet ring cells, any extracellular mucin component; Group 2 ≤ 50% signet ring cells, <50% extracellular mucin; Group 3 ≤ 50% signet ring cells, ≥50% extracellular mucin.

The 5-year OS was 34% (95% CI 22–53) in the patients with SRC tumours and 55% (95% CI 55–60) in the patients with non-SRC tumours (*p* < 0.001, Figure 4a). When analysing the patients with information available on the extracellular mucin status, both AC (*n* = 1397) and MAC (*n* = 215) had better 5-year OS rates at 70% (95% CI 68–73) and 62% (95% CI 58–73), respectively, compared to the SRC tumour patients (*p* < 0.001, Figure 4b). Figure 4c illustrates the impact of the SRC component on OS. The patients with SRC ≥ 50 had a 5-year OS rate of 20% (95% CI 6–70), and those with SRC < 50 had a 5-year OS rate of 39% (95% CI 24–61) (*p* < 0.001). When including information on extracellular mucin, Group 1 (SRC ≥ 50) patients had a 5-year OS rate of 20% (95% CI 6–70), Group 2 (SRC< 50, <50% extracellular mucin) had a 5-year OS rate of 34% (95% CI 19–61), and Group 3 (SRC < 50, ≥50% extracellular mucin) had a 5-year OS rate of 50% (95% CI 25–99, *p* < 0.001, Figure 4d). There were no statistically significant OS differences between AC, SRC≥ 50, and SRC< 50, in stage II, stage III, or stage IV patients (File S2).

### 3.6. Recurrence-Free Survival

**Figure 5:**
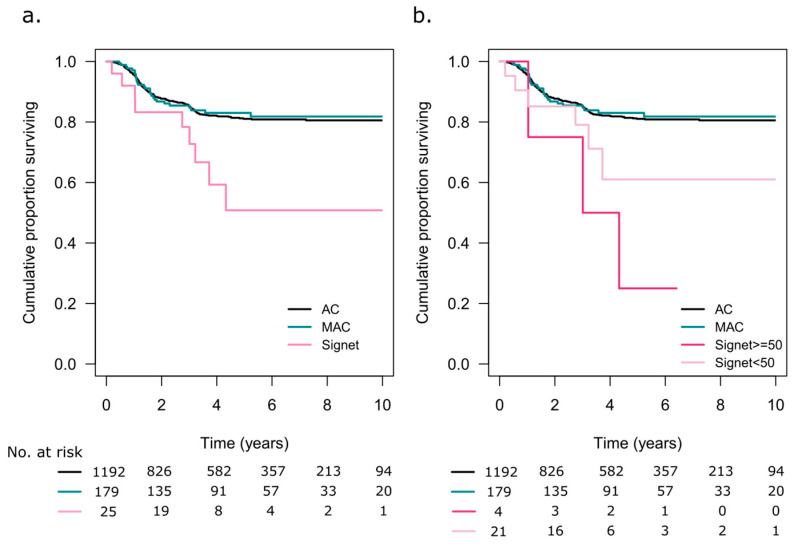
(**a**) Recurrence-free survival in patients with adenocarcinoma (AC), mucinous adenocarcinoma (MAC), and signet ring cell tumours. Only patients with information on extracellular mucin were included. (**b**) Patients with signet ring cell tumours were categorized based on signet ring cells comprising ≥50% or <50% of the tumour tissue.

RFS in stage I–III patients undergoing resection surgery with curative intent is shown in Figure 5a. The patients with SRC tumours had the poorest 5-year RFS rate at 51% (95% CI 13–83), compared to AC with 81% (95% CI 79–84) and MAC, 83% (95% CI 77–89, *p* = 0.02). Only 4 patients with SRC ≥ 50 had underwent resection with curative intent, and 1 (25%) was recurrence free at 5 years, while patients with SRC < 50 had a 5-year RFS rate of 61% (95% CI 40–94, *p* = 0.008, Figure 5b).

### 3.7. Risk Factors for Recurrence

Risk factors for recurrence in stage I–III colon cancer are shown in Table 3. In a univariate analysis, SRC ≥ 50 was a risk factor (HR 4.91, 95% CI 1.56–15.45), but pT4, pN1/N2, vascular, and perineural invasion were the only independent risk factors in the multivariate analysis. In all (including the patient with rectal cancer), 9 (36%) patients with SRC tumours had recurrence, with all but 2 having a peritoneal recurrence.

### 3.8. Appendiceal Tumours

A subgroup analysis was performed on the appendiceal tumours; the baseline clinical and histopathological characteristics are shown in File S3. A total of 18 appendiceal cancers (0.8% of the whole cohort) were included in the SCRCR, of which 8 (44%) had SRCs. Patients with SRC tumours tended to have more advanced disease, which was not amenable for resection, and had a worse OS than those with non-SRC appendiceal cancer (*p* = 0.03).

## 4. Discussion

In this study, a consecutive cohort of histopathologically verified SRC colorectal and appendiceal cancers were found to have aggressive clinical and histopathological features, with no patient being diagnosed as stage I and more than half being diagnosed as stage IV. In addition, all the adverse histopathological features—high-grade tumours, vascular invasion, and perineural invasion—were more common in SRC tumours. SRCs were associated with a unique pattern of metastasis, with the peritoneum being the most common site. Most importantly, patients with SRC ≥ 50 had the poorest OS and RFS rates, and patients with SRC < 50 also had impaired prognosis and aggressive histopathological features.

The prevalence of SRC ≥ 50 varies but has been reported to be 0.6–1.1% [3,4,6,8,9,10,11,12]. In the present study, SRC ≥ 50 was rare, with a confirmed prevalence of 0.45%. If the patients with SRC < 50 were included, of whom many had a SRC component of ~40%, the prevalence was 2.3%. The low prevalence of SRC ≥ 50 in our study might have been due to the thorough re-examination and the application of a strict ≥50% criteria or to differences between the pathologists assessing the proportion of SRCs. If the original pathologist did not mention ‘signet’ in the histopathology report, our free-text search method would have failed to identify that patient. However, the presence of SRCs, especially those ≥50%, is routinely mentioned, and these patients should have been identified. In addition, preoperative biopsies and biopsies from metastases could have been sampled from non-representative tumour tissue. However, despite the low prevalence, with 6500 colorectal cancers being diagnosed in Sweden annually (out of 10 million inhabitants, 2020), the estimated incidence of SRC-containing colorectal cancer is about 150 cases/year [29].

The SRC tumours had a right-sided distribution, with most tumours localized in the cecum and the ascending colon; only two patients had SRC tumours located in the rectum. This confirmed previous reports [3,4,6,7,8,9,10,11,12]. Appendiceal adenocarcinomas (not neuroendocrine) were also included in the SCRCR, and the appendix was a common site for SRC tumours. Studies on SRC appendiceal tumours have been scarce due to the rarity of these tumours, but a higher proportion of SRC tumours in the appendix, than in other sites, in the colon and rectum has been reported [30]. SRC ≥ 50 has been associated with younger age and male predominance, but this association was not significant when analysing all patients with SRC tumours. However, the patients with SRC ≥ 50 tended to be younger and more often male. Confirmed hereditary colorectal cancer was not common, but the results indicated a high incidence of prior malignancies in patients with SRC tumours. Comparable data for patients with non-SRC tumours were not available.

SRCs have been strongly associated with peritoneal metastases [7,17,31,32], and this was confirmed in the present study. No SRC patients had synchronous lung metastases. This strengthens the suspicion of SRCs as a marker of a unique biological behaviour, rather than as a marker of poor differentiation. The molecular profile of SRC tumours is unclear, as is the mechanism of peritoneal dissemination. It has been suggested that SRC ≥ 50 comprise 2 subgroups, 1 hypermethylated/microsatellite-unstable/BRAF-mutated in the proximal colon and 1 hypomethylated in the distal colon [22]. The first group could be a potential target for immune checkpoint inhibitors [22,23].

The patients with SRC tumours had a poorer survival rate, as compared to the non-SRC tumour patients. Contrary to what had previously been reported [6], there were no significant survival differences in stage II, stage III, and stage IV, which would suggest that the poor survival of SRC patients is mainly due to an increased ability of the tumours to metastasize. There was, however, a tendency towards poorer prognoses in patients with SRC tumours, though the patient number was low. Despite SRC ≥ 50 being the histopathological subgroup with the poorest prognosis [3,4,6,7], it is not an official risk factor and, hence, not registered in the SCRCR. Often, studies have drawn a strict line between ≥50% and <50% SRCs. Here, and in some other studies [7,17,18,19,20,21], the patients with SRC < 50 had poorer prognosis. In addition, the extracellular mucin component appeared to have a positive prognostic impact. In all, the SRC component should be routinely registered in pathology reports and clinical registers in order to enable larger studies that can aid our understanding of its prognostic role.

This study had several limitations. First, SRCs were not reported to the SCRCR. The free-text search in the hospital’s data system for the histopathology reports was not validated, and therefore, patients may have been overlooked. However, to our knowledge, this was the safest procedure for identifying the patients, aside from re-examining all the histological slides. Second, the analyses were based on register data, and differences in reporting to the registry or in interpreting the data might have existed. Regarding metastases, before 2017, all sites of metastases other than liver and lung metastases were based on a free-text description. However, the registration was limited to one hospital and a limited number of people, decreasing its variability. Furthermore, as the hospital is a centre for cytoreductive surgery, liver surgery, and thoracic surgery, the risk of failing to report metastases accurately to the registry should have been low. Regarding the extracellular mucin, non-SRC patients without data on this were excluded from OS analyses separating AC and MAC, which likely led to the exclusion of some stage IV patients. However, this bias was not present in the RFS analyses, where the same differences in OS were found. Lastly, as for other studies on SRCs, the small number of patients limited the ability to compare the subgroups, but the poor prognosis associated with SRC ≥ 50 and SRC < 50 were clear.

## 5. Conclusions

The presence of SRCs in colorectal and appendiceal cancer was associated with aggressive histopathological features, advanced stages, and, particularly, peritoneal metastases. These patients had the poorest OS and RFS among all colorectal and appendiceal cancer patients. Information about the presence of SRCs in tumour tissue, not only in the case of ≥50% SRCs, should be routinely registered in pathology reports and clinical registers.

## Figures and Tables

**Table 1:** Clinical characteristics of patients with colorectal and appendiceal cancer 2009–2020 in Uppsala, Sweden (*n* = 2229). Patients were divided based on the presence of signet ring cells (Signet) or absence of signet ring cells (Non-Signet).

Clinical Characteristics	Signet	Non-Signet	*p*-Value
	*n* = 51	*n* = 2178	
	*n* (%)	*n* (%)	
Sex			0.507
Male	24 (47)	1149 (52.8)	
Female	27 (53)	1029 (47.2)	
Age, years, median (IQR)	70 (64–79)	72 (64–79)	0.472
Age groups			0.524
<50	2 (8)	104 (5)	
50–59	3 (6)	220 (10)	
60–69	18 (35)	587 (27)	
70–79	14 (27)	753 (35)	
80–89	11 (22)	442 (20)	
≥90	1 (2)	72 (3)	
Localisation			<0.001
Appendix	8 (16)	10 (0.5)	
Colon			
Caecum	10 (20)	353 (16)	
Ascending	11 (22)	224 (10)	
Hepatic flexure	2 (4)	106 (5)	
Transverse	7 (14)	109 (5)	
Splenic flexure	1 (2)	71 (3)	
Descending	1 (2)	63 (3)	
Sigmoid	5 (10)	503 (23)	
Rectum	2 (4)	735 (34)	
Colorectum NOS	4 (8)	4 (0.2)	
^1^ Stage			<0.001
I	0 (0)	291 (13)	
II	8 (16)	491 (23)	
III	17 (33)	822 (38)	
IV	26 (51)	515 (24)	
Missing information	0 (0)	59 (2.7)	
Emergency surgery	11 (22)	259 (12)	0.06
Synchronous/metachronous			0.01
Synchronous	1 (2)	28 (1)	
Metachronous	3 (6)	19 (1)	

^1^ Pathological staging. Clinical staging was used in cases of neoadjuvant treatment or if pathological staging was not available. IQR: interquartile range. NOS: not otherwise specified.

**Table 2:** Histopathological characteristics of curatively resected colon and appendiceal cancer in 2009–2020 in Uppsala, Sweden (*n* = 1176). Patients were divided based on the presence of signet ring cells (Signet) or absence of signet ring cells (Non-Signet). Data from the Swedish Colorectal Cancer Registry.

Clinical Characteristics	Signet	Non-Signet	*p*-Value
	*n* = 37	*n* = 1139	
	*n* (%)	*n* (%)	
Tumour stage			<0.001
T1	0 (0)	78 (7)	
T2	0 (0)	116 (10)	
T3	12 (32)	661 (58)	
T4	24 (65)	284 (25)	
TX	1 (3)	0 (0)	
Node stage			<0.001
N0	7 (19)	597 (52)	
N1	8 (22)	310 (27)	
N2	20 (54)	227 20)	
NX	2 (5)	3 (0.3)	
Missing information	0 (0)	2 (0.3)	
Vascular invasion			<0.001
Yes	26 (70)	360 (32)	
No	6 (14)	731 (64)	
Missing information	5 (16)	48 (4)	
Perineural invasion			<0.001
Yes	14 (38)	221 (19)	
No	18 (49)	861 (76)	
Missing information	5 (14)	57 (5)	
Differentiation grade			<0.001
High	29 (78)	209 (18)	
Low	7 (19)	890 (78)	
Missing information	1 (3)	40 (4)	
^1^ Mucinous			<0.001
Yes	15 (41)	173 (15)	
No	22 (59)	926 (81)	
Missing information	0 (0)	40 (4)	

^1^ Mucinous ≥ 50% extracellular mucin. A mucinous component < 50% was included in ‘No’ category.

**Table 3:** Univariate and multivariate Cox proportional regression hazard analyses for risk of recurrence in patients with colon cancer (including appendiceal cancer) curatively resected with mesocolic resection and central ligation (*n* = 1019).

Clinical and Histopathological Characteristics	Univariate	^1^ Multivariable	Multivariable *p*-Value
	HR (95% CI)	HR (95% CI)	
Sex			
Male	1.00		
Female	1.10 (0.80–1.51)		
Age	0.99 (0.98–1.01)		
Localisation			
Appendix	2.28 (0.56–9.36)	2.62 (0.53–13.00)	
Right colon	**1.44 (1.03–2.02)**	1.00	
Left colon	1.00	1.28 (0.90–1.82)	
Colon NOS	0.00 (0.00– )	0.00 (0.00– )	
Preoperative treatment			
No preoperative treatment	1.00		
Chemotherapy	1.33 (0.19–9.36)		
Radiotherapy	1.08 (0.51–2.31		
Chemoradiotherapy	0.00 (0.00– )		
Surgery			
Open	1.00		
Laparoscopy	**0.48 (0.31–0.74)**		
Converted	0.64 (0.33–1.27)		
Adjuvant treatment			
No	1.00		
Yes	**2.06 (1.46–2.92)**		
pT stage			
T1	0.00 (0.00– )	0.00 (0.00– )	
T2	1.00	1.00	
T3	**2.33 (1.02–5.35)**	1.36 (0.58–3.18)	
T4	**6.86 (2.98–15.79)**	**2.56 (1.06–6.17)**	0.036
pN stage			
N0	1.00	1.00	
N1	**2.74 (1.79–4.19)**	**1.79 (1.14–2.80)**	0.011
N2	**7.08 (4.76–10.54)**	**3.55 (2.26–5.57)**	<0.001
Histopathology			
AC	1.00	1.00	
MAC	0.88 (0.55–1.42)	1.22 (0.69–1.84)	
Signet ring ≥ 50%	**4.91 (1.56–15.45)**	1.40 (0.43–4.61)	
Signet ring < 50%	1.83 (0.75–4.47)	0.72 (0.26–2.05)	
Vascular invasion			
Yes	**3.69 (2.66–5.13)**	**1.72 (1.18–2.52)**	0.005
No	1.00	1.00	
Perineural invasion			
Yes	**3.10 (2.19–4.37)**	**1.57 (1.08–2.29)**	0.019
No	1.00	1.00	
Differentiation grade			
High	**2.19 (1.55–3.10)**	1.17 (0.79–1.73)	
Low	1.00	1.00	

^1^ Statistically significant variables in the univariate analyses, except treatment variables, were included in the multivariate analysis. HR: hazard ratio; CI: confidence interval; NOS: not otherwise specified; pT: pathological tumour stage; pN: pathological node stage; AC: adenocarcinoma; MAC: mucinous adenocarcinoma.

## Data Availability

The data presented in this study are not publicly available according to the ethics committee, as the data contains personal information.

## Supplementary Materials

The following supporting information can be downloaded at: https://www.mdpi.com/article/10.3390/cancers15092497/s1, File S1: Clinico-histopathological characteristics of signet ring cell tumours; File S2: Stage specific overall survival; File S3: Appendiceal cancer.
