# Supplementary material for: Signet Ring Cell Colorectal and Appendiceal Cancer: A Small Signet Ring Cell Component Is Also Associated with Poor Outcome
Source: Cancers (Basel). 2023 Apr 26;15(9):2497. doi: 10.3390/cancers15092497 (PMC10177230; doi:10.3390/cancers15092497)
Supplement: Supplementary file 1 [file cancers-15-02497-s001.zip › File S1 Signet tables revision.pdf]

Supplementary Table S1a. Clinical characteristics of patients with colorectal and appendiceal cancer and signet ring cells, 2009–2020, in Uppsala, Sweden (n= 51). Patients are divided based on signet ring cell and extracellular mucin components.

Characteristics	**Group 1**<br>≥ 50% signet, any mucin<br>n = 10	**Group 2**<br>< 50% signet, < 50% mucin<br>n = 28	**Group 3**<br>< 50% signet, > 50% mucin<br>n = 13
	n (%)	n (%)	
Sex			
Male	6 (60)	10 (36)	8 (62)
Female	4 (40)	18 (64)	5 (38)
Age, years, median (IQR)	66 (63–70)	74 (66–79)	68 (61–81)
Age groups			
< 45	0 (0)	0 (0)	2 (15)
45 - 49	0 (0)	1 (4)	1 (8)
50 -59	2 (20)	0 (0)	1 (8)
60-69	4 (40)	10 (36)	4 (31)
70-79	3 (30)	10 (36)	1 (8)
80-89	1 (10)	6 (21)	4 (31)
≥90	0 (0)	1 (4)	0 (0)
Localisation			
Appendix	1 (10)	6 (21)	1 (8)
Colon			
Cecum	2 (20)	6 (21)	2 (15)
Ascending	3 (30)	5 (18)	3 (23)
Hepatic flexure	1 (10)	0 (0)	1 (8)
Transverse	0 (0)	5 (18)	2 (15)
Splenic flexure	0 (0)	0 (0)	1 (8)
Descending	0 (0)	0 (0)	1 (8)
Sigmoid	2 (20)	2 (7)	1 (8)
Rectum	0 (0)	1 (4)	1 (8)
Colorectum NOS	1 (10)	3 (11)	0 (0)
[a]Stage			
I	0 (0)	0 (0)	0 (0)
II	0 (0)	5 (18)	3 (23)
III	4 (40)	7 (25)	6 (46)
IV	6 (60)	16 (57)	4 (31)

[a]Pathological staging. Clinical staging was used in cases of neoadjuvant treatment or if pathological staging was not available, for example in palliative patients.

IQR: interquartile range

NOS: not otherwise specified

Supplementary Table S1b. Histopathological characteristics of patients with signet ring cells with available information on pathological characteristics 2009–2020 in Uppsala, Sweden (n = 51). Patients are divided based on signet ring cell and extracellular mucin components. Missing information varies depending on if resection was done or not.

Characteristics	**Group 1**<br>≥ 50% signet, any mucin<br>n = 10	**Group 2**<br>< 50% signet, < 50% mucin<br>n = 28	**Group 3**<br>< 50% signet, > 50% mucin<br>n = 13
	n (%)	n (%)	
pTumour stage			
T1	0 (60)	0 (0)	0 (0)
T2	0 (40)	0 (0)	0 (0)
T3	1 (40)	7 (25)	5 (38)
T4	6 (40)	13 (46)	6 (46)
TX (uncertain)	1 (10)	4 (14)	1 (8)
Missing information	2 (20)	4 (14)	2 (15)
pNode stage			
N0	0 (0)	6 (21)	2 (15)
N1	2 (20)	5 (18)	2 (15)
N2	5 (50)	9 (32)	6 (46)
NX (uncertain)	1 (10)	4 (14)	2 (15)
Missing information	2 (20)	4 (14)	1 (8)
Vascular invasion			
Yes	6 (60)	13 (46)	7 (54)
No	1 (10)	3 (11)	3 (23)
Missing information	2 (20)	12 (43)	3 (23)
Perineural invasion			
Yes	3(30)	9 (32)	2 (15)
No	4 (40)	7 (25)	8 (62)
Missing information	3 (30)	12 (43)	3 (23)
Differentiation grade			
High	5 (50)	17 (61)	9 (69)
Low	1 (10)	4 (14)	2 (15)
Missing information	4 (40)	7 (25)	2 (15)
