# Supplementary material for: Signet Ring Cell Colorectal and Appendiceal Cancer: A Small Signet Ring Cell Component Is Also Associated with Poor Outcome
Source: Cancers (Basel). 2023 Apr 26;15(9):2497. doi: 10.3390/cancers15092497 (PMC10177230; doi:10.3390/cancers15092497)
Supplement: Supplementary file 1 [file cancers-15-02497-s001.zip › Supplementary material File S3.pdf]

Supplementary Table. Clinical characteristics of patients with appendiceal cancer 2009–2020 in Uppsala, Sweden (n = 18). Patients are divided based on presence of signet ring cells (Signet) or absence of signet ring cells (Non-Signet).

Clinical characteristics	Signet n = 8	Non-Signet n = 10
	n (%)	n (%)
Sex		
Male	3 (38)	7 (70)
Female	5 (63)	3 (30)
Age, years, median (IQR)	71 (66–76)	63 (60–70)
Type of surgery		
Appendectomy	0 (0)	1 (10)
Ileocecal resection	0 (0)	3 (30)
Right hemicolectomy	2 (25)	5 (50)
Other	2 (25)	1 (10)
No surgery	4 (4)	0 (0)
Emergency surgery		
Yes	1 (13)	4 (40)
No	7 (86)	6 (60)
[a]Stage		
I	0 (0)	0 (10)
II	1 (13)	2 (20)
III	1 (13)	1 (10)
IV	6 (75)	5 (50)
Missing information	0 (0)	1 (10)
Mucinous		
Yes	1(13)	5 (50)
No	7 (88)	5 (50)
Vascular invasion		
Yes	1 (13)	1(10)
No	0 (0)	9 (90)
Missing information	7 (88)	
Perineural invasion		
Yes	0 (0)	1(10)
No	0 (0)	9 (90)
Missing information	8 (100)	0 (0)
Differentiation grade		
High	4 (50)	1(10)
Low	1 (13)	9 (90)
Missing information	3 (38)	0 (0)

[a]Pathological staging. Clinical staging was used in cases of neoadjuvant treatment or if pathological staging was not available

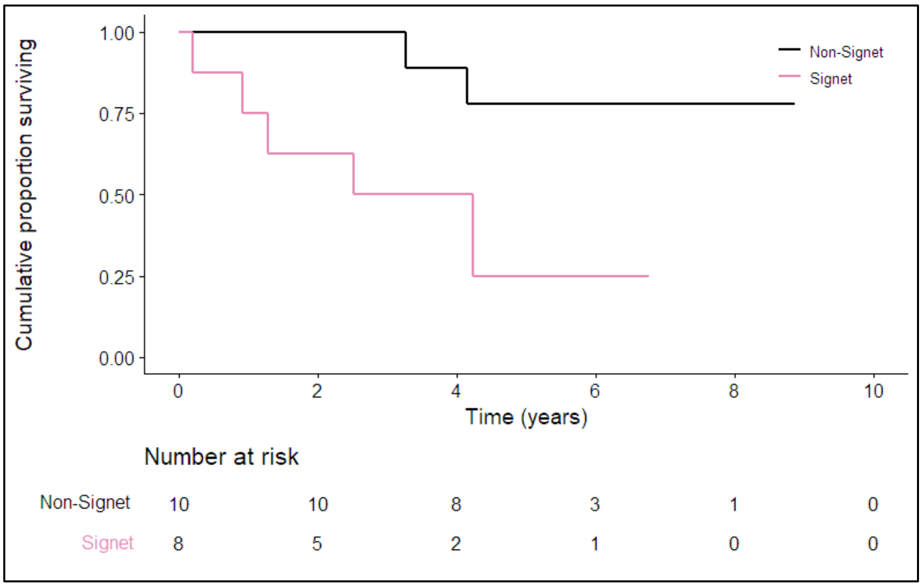

Supplementary Figure. Overall survival for patients with appendiceal tumours. Patients are separated based on presence of signet ring cells (Signet) or no presence of signet ring cells (Non-Signet).
