# Supplementary figures and images for: Signet Ring Cell Colorectal and Appendiceal Cancer: A Small Signet Ring Cell Component Is Also Associated with Poor Outcome
Source: Cancers (Basel). 2023 Apr 26;15(9):2497. doi: 10.3390/cancers15092497 (PMC10177230; doi:10.3390/cancers15092497)

## Stage II

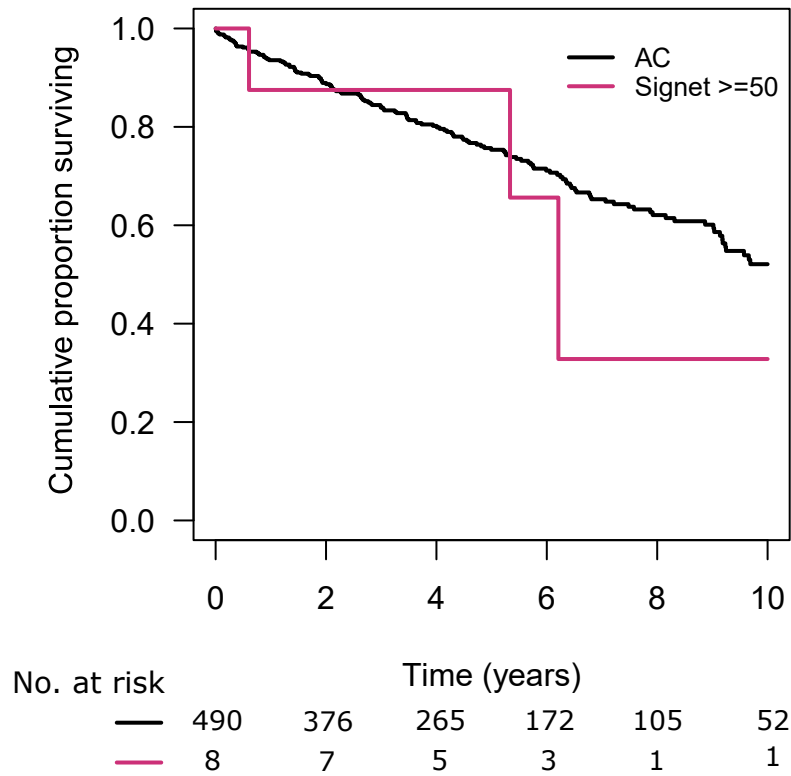

## Stage III

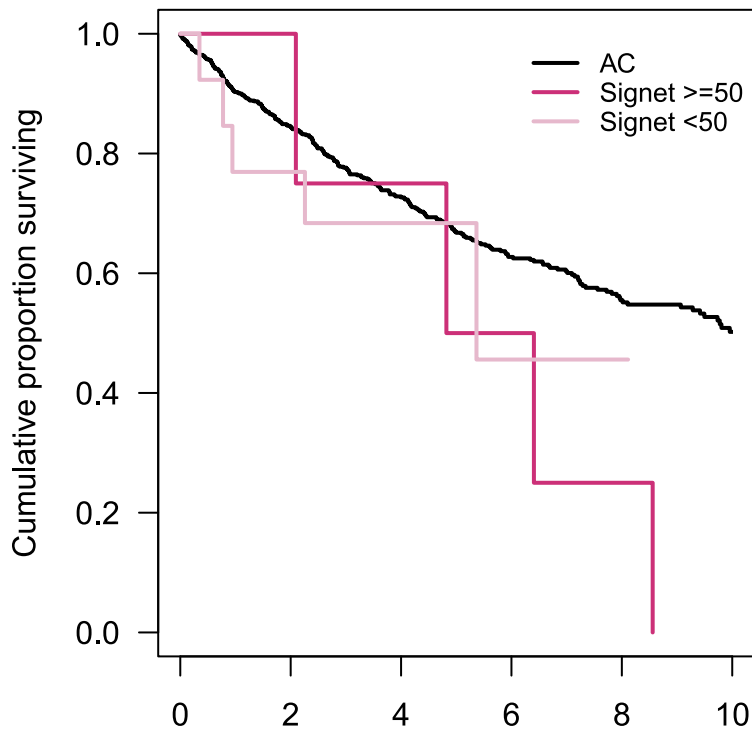

## Stage IV

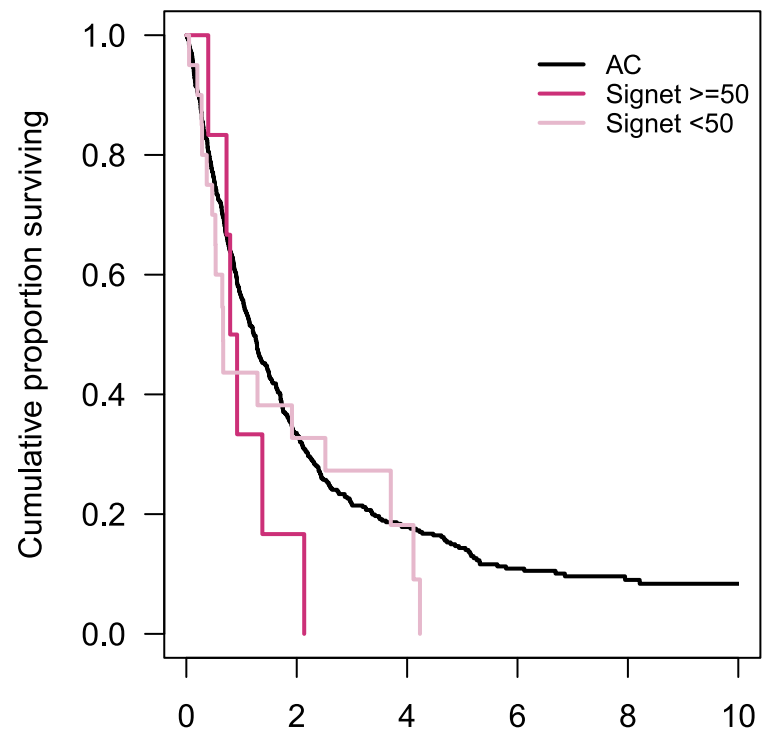

Supplement: Supplementary file 1 [file cancers-15-02497-s001.zip › File S2 stage OS.pdf]
